# Alterations of the Fatty Acid Profile and the Expression of Genes Related to FA Metabolism in Cirrhotic Liver Tissue

**DOI:** 10.3390/ijms25158115

**Published:** 2024-07-25

**Authors:** Aleksandra Hliwa, Oliwia Lange-Andrzejewska, Dariusz Laski, Maciej Sledzinski, Piotr Remiszewski, Anna Drobinska, Adriana Mika, Tomasz Sledzinski

**Affiliations:** 1 Department of Pharmaceutical Biochemistry, Faculty of Pharmacy, Medical University of Gdansk, Debinki 1, 80-211 Gdansk, Poland; aleksandra.hliwa@gumed.edu.pl (A.H.); tsledz@gumed.edu.pl (T.S.); 2 Department of Environmental Analytics, Faculty of Chemistry, University of Gdansk, Wita Stwosza 63, 80-308 Gdansk, Poland; oliwia.lange@phdstud.ug.edu.pl; 3 Department of General, Endocrine and Transplant Surgery, Faculty of Medicine, Medical University of Gdansk, Smoluchowskiego 17, 80-214 Gdansk, Poland; dariusz.laski@gumed.edu.pl (D.L.); msledz@gumed.edu.pl (M.S.); piotr.remiszewski@gumed.edu.pl (P.R.); 4 Department of Paediatrics, Nephrology and Hypertension, Faculty of Medicine, Medical University of Gdansk, Smoluchowskiego 17, 80-214 Gdansk, Poland; anna.drobinska@gumed.edu.pl

**Keywords:** cirrhosis, fatty acids, ALD, NASH, NAFLD, lipids

## Abstract

In addition to direct damage to hepatocytes, long-term ethanol consumption leads to lipid accumulation and hepatic steatosis, as well as to the dysregulation of lipid metabolism. The final step in various liver diseases is cirrhosis. The aim of this study was to compare the FA (fatty acids) profile and expression levels of genes involved in lipid metabolism in cirrhotic liver tissue and normal liver tissue. Exploring the changes in the FA profile and expression of genes related to fatty acid metabolism in cirrhotic liver tissue reveals a molecular landscape that goes beyond the surface of traditional liver function assessments. Understanding the shifts in gene expression and fatty acid composition in liver tissue opens avenues for interventions that may aid in the treatment of cirrhosis in the future.

## 1. Introduction

Liver cirrhosis is caused by the progressive formation of scar tissue, which leads to impaired liver function. This damage usually occurs as a result of non-alcoholic fatty liver disease (NAFLD), alcoholic liver disease (ALD), hepatitis B (HBV) or C (HCV) infection, or autoimmune diseases. ALD is responsible for almost half of cirrhosis-related deaths (47.9%) worldwide [1]. Despite the global trend showing a significant increase in NAFLD in the general population and in patients with liver disease, the majority of patients in our clinic and in Poland in general are still diagnosed with cirrhosis due to alcohol abuse. ALD can be diagnosed when the accumulation of fat in the liver is associated with excessive and regular alcohol consumption—more than 30 g/day (3 drinks) for men and more than 20 g/day (2 drinks) for women [2,3]. In addition to direct damage to hepatocytes, long-term ethanol consumption leads to lipid accumulation and hepatic steatosis, as well as to the dysregulation of lipid metabolism. It is described that ethanol leads to an imbalance in fatty acid (FA) composition by upregulating FA uptake and downregulating the action of lipid transporters that enable lipid export from the liver to the circulatory system [4].

The prevalence of chronic liver disease is increasing worldwide, with NAFLD being the most common. NAFLD also includes non-alcoholic steatohepatitis (NASH), which is characterized by the inflammation and death of hepatocytes. NASH can lead to the development of fibrosis, cirrhosis, and even hepatocellular carcinoma [5]. The prevalence of NAFLD in Western Europe is around 25% [6]. However, as there are no non-invasive tests for early detection, these figures may be underestimated. Diagnosis is based on the result of the histopathological examination of a liver biopsy, which is performed when significant symptoms are present and therefore at a high stage of the disease [5]. The development of NAFLD is closely associated with insulin resistance, type 2 diabetes mellitus (T2D), hypertension, and obesity. Therefore, the prevalence of NAFLD in patients with T2D is probably even higher than indicated above. It has been proposed that NAFLD’s name should be changed to metabolic-associated fatty liver disease (MAFLD) to reflect the manifestations of systemic metabolic disturbances due to the accumulation of fat in the liver [7].

The liver is an essential organ of lipid metabolism; however, the accumulation of lipids can lead to alterations in the metabolic pathways involved in the synthesis of FAs [8]. FAs are the basic components that make up most lipid classes and determine their properties. Previous lipidomic studies suggest changes in the FA profile of the liver within ALD and NAFLD patients, including an increase in the level of saturated fatty acids (SFAs), monounsaturated fatty acids (MUFAs), and polyunsaturated fatty acids (PUFAs) [9,10]. In particular, SFAs and PUFAs were found in higher concentrations in NAFLD compared to healthy livers [9]. This also indicates an increased expression of lipid synthesis and desaturation enzymes [10]. In contrast, many studies show a particular decrease in long-chain PUFAs in the liver as a result of long-term alcohol consumption [11]. These changes may be crucial for the development of liver cirrhosis [5].

The final step in various liver diseases is cirrhosis. The FA profile and metabolism in the cirrhotic liver have not yet been studied in detail. Thus, the aim of this study was to compare the FA profile and expression levels of genes involved in lipid metabolism in cirrhotic liver tissue and normal liver tissue. To identify the molecular mechanisms of the above-mentioned lipid changes, the expression of genes related to lipid metabolism was also examined in normal and cirrhotic liver tissue samples.

## 2. Results

### 2.1. Effect of Cirrhosis on Hepatic FA Profile

**Figure 1:**
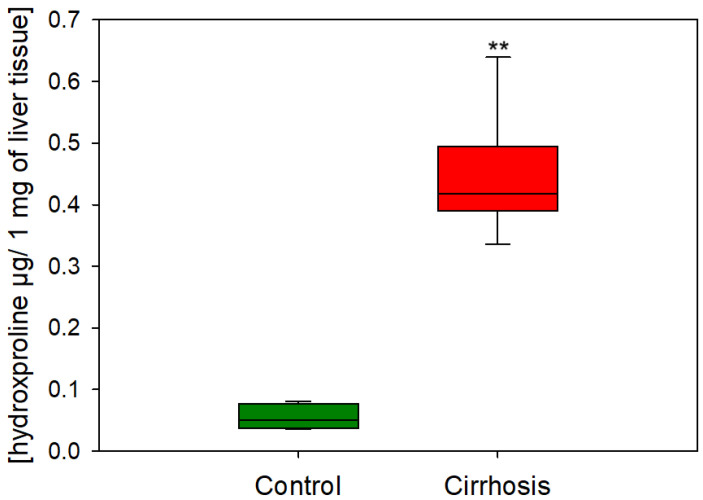
Content of hydroxyproline in µg per 1 mg of liver tissue in hepatic control group and cirrhotic liver tissue. ** *p* < 0.0001.

To examine the progression of liver fibrosis, we analyzed the hydroxyproline concentration [12] in cirrhotic tissue and in control samples. We found strongly elevated levels of hydroxyproline in cirrhotic tissue (Figure 1).

**Figure 2:**
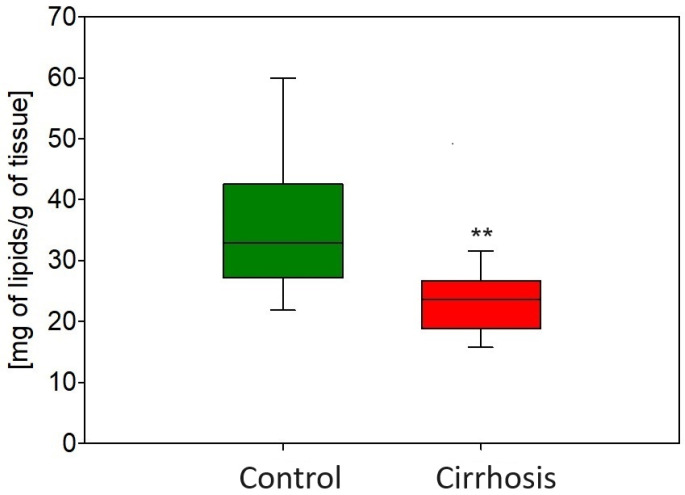
Content of total lipids in mg per 1 g of liver tissue in the hepatic control group and cirrhotic liver tissue. ** *p* < 0.001.

In cirrhotic tissue, where the fibrosis process is advanced and fibrin is deposited, the lipids make up a lower proportion of the mass per 1 g of tissue (Figure 2).

We also found an interesting inverse correlation between the lipid mass per 1 g of tissue and the MELD-Na score (R = −0.586, *p* = 0.012). The MELD-Na scale (Model of End-Stage Liver Disease) is a clinical imaging tool used to assess liver tissue destruction. The MELD-Na scale indicates the severity of a patient’s condition and the urgency of transplantation, with higher numerical values indicating a worse condition. In our study, patients with cirrhosis had higher MELD-Na scores and lower lipid content per gram of tissue compared to the hepatic control group (Table 1).

**Figure 3:**
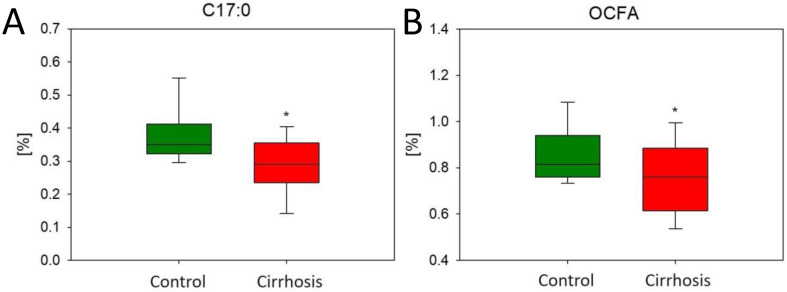
Content of C17:0 (**A**) and OCFA (**B**) in the hepatic control group (Ctrl) and cirrhotic liver tissue. * *p* < 0.05.

We then analyzed the FA profiles in control and cirrhotic liver samples. We found an increased level of heptadecanoic acid (C17:0) (0.29% ± 0.018 vs. 0.37% ± 0.021, *p* < 0.05) in the cirrhotic livers compared to the normal liver samples. The same trend was also observed in the whole group of odd-chain fatty acids (OCFAs) (Figure 3).

**Figure 4:**
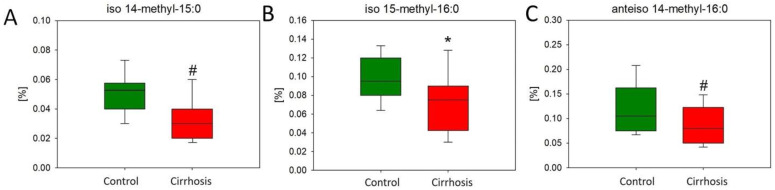
Content of iso 14-methyl-15:0 (**A**), iso 15-methyl-16:0 (**B**), and anteiso 14-methyl-16:0 (**C**) in the hepatic control group and cirrhotic liver tissue. For non-parametric values: Mann–Whitney U test # *p* < 0.05; for parametric: Student’s *t*-test * *p* < 0.05.

Similar to the OCFA content, lower levels of individual representatives of branched-chain fatty acids (BCFAs) were found in the cirrhotic tissue sections compared to the hepatic control tissue samples. In Figure 4, we have shown three of them that reached statistical significance in the comparison between groups.

**Figure 5:**
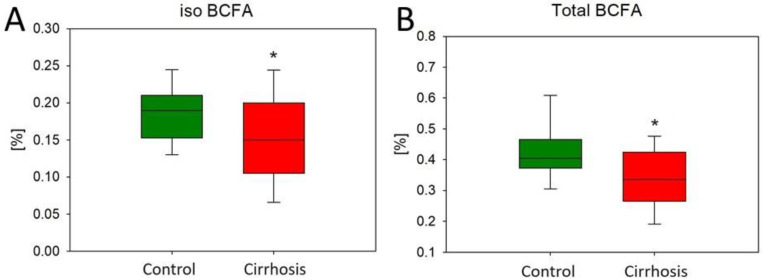
Content of total iso BCFAs (**A**) and total BCFAs (**B**) in the hepatic control group and cirrhotic liver tissue. * *p* < 0.05.

In addition, we found significantly lower levels of total BCFAs in the tissues of patients with cirrhosis compared to normal tissues. Most of them showed iso conformation (total iso BCFAs). These two results can be seen in the graphs below (Figure 5).

**Figure 6:**
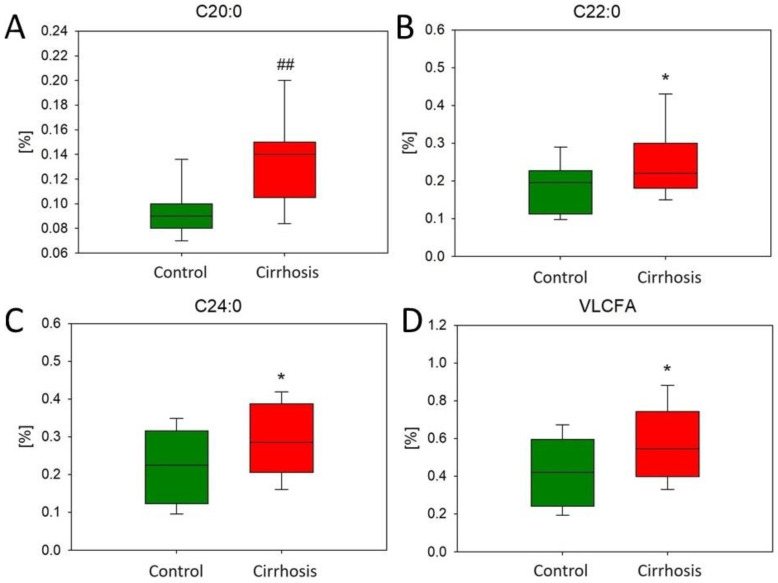
Content of various VLC-SFA (**A**–**C**) and total VLC-SFA (**D**) in the hepatic control group and cirrhotic liver tissue. For non-parametric values: Mann–Whitney U test ## *p* < 0.001; for parametric: Student’s *t*-test * *p* < 0.05.

Our study revealed a statistically significant increased amount of saturated very long-chain fatty acids (VLCFAs) with 20–24 carbon atoms in tissues from patients with cirrhosis compared to tissues from the hepatic control group (Figure 6).

**Figure 7:**
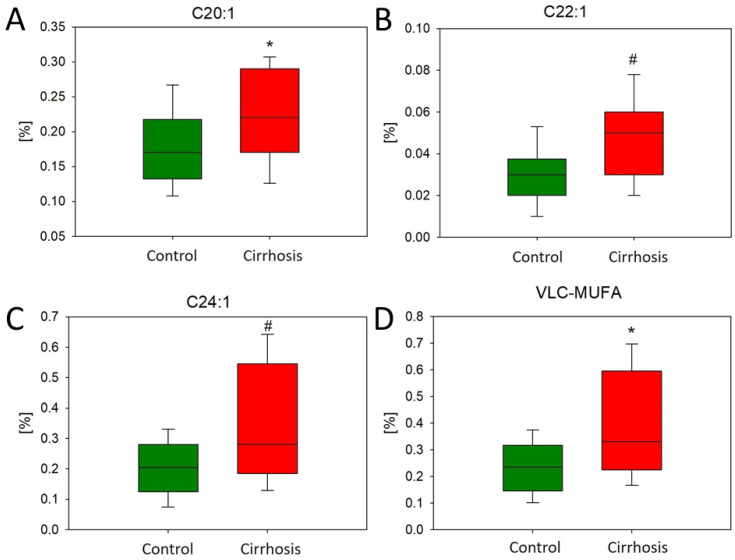
Content of representatives (**A**–**C**) and total VLC-MUFAs (**D**) in hepatic control group and cirrhotic liver tissue samples. For parametric values: Student’s *t*-test * *p* < 0.05; for non-parametric: Mann–Whitney U test # *p* < 0.05.

Furthermore, monounsaturated VLCFAs (VLC-MUFAs) at 20, 22, and 24 carbons were significantly higher in the tissue samples of patients with liver cirrhosis than in the tissue samples of the hepatic control (Figure 7).

**Figure 8:**
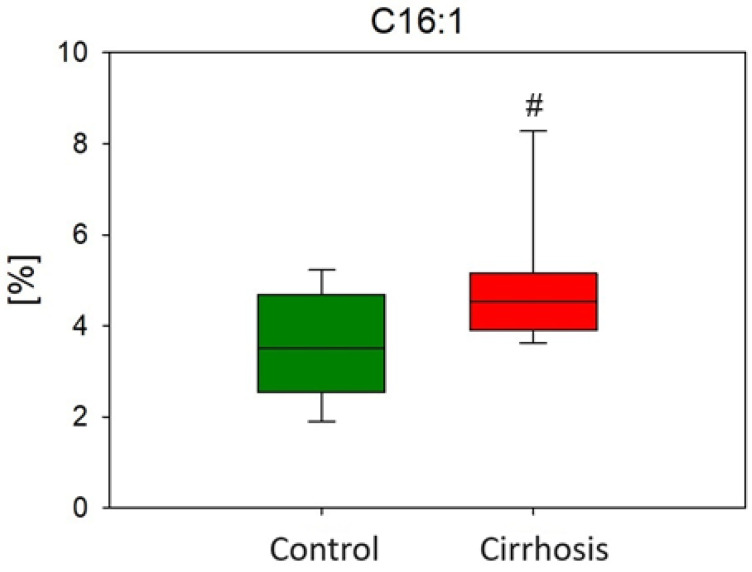
Content of C16:1 in hepatic control group and cirrhotic liver tissue. Mann–Whitney U test # *p* < 0.05.

In addition, the level of palmitoleic acid (C16:1) was higher in cirrhotic samples compared to normal tissue samples (*p* = 0.02) (Figure 8). This may have been a result of increased desaturation in the cirrhosis liver samples compared to hepatic control samples (0.20 ± 0.073, 0.15 ± 0.046, respectively, *p* = 0.008).

**Figure 9:**
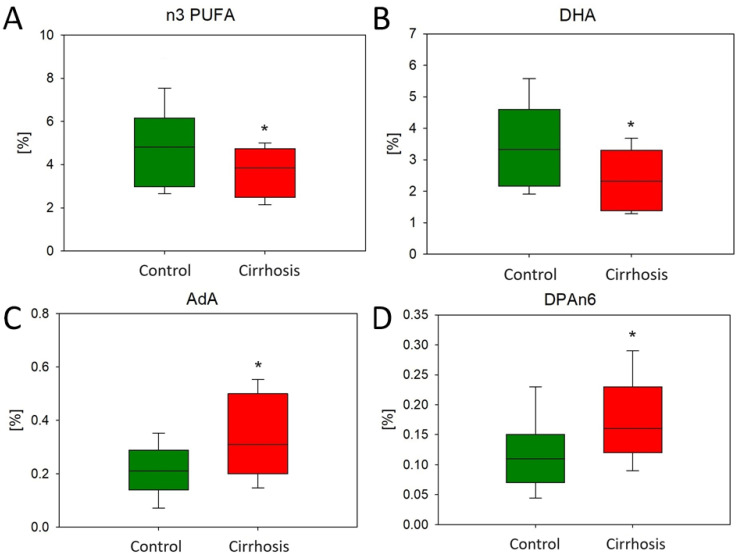
Content of DHA (**A**), total n3 PUFAs (**B**), AdA(**C**), and n6 DPA (**D**) in the hepatic control group and cirrhotic liver tissue. * *p* < 0.05.

We found reduced levels of n3 FAs in patients with cirrhosis. Furthermore, the level of docosahexaenoic acid (DHA), which is the most important among the n3 PUFAs, was significantly lower in the cirrhotic tissues compared to the tissues of the hepatic control (Figure 9). On the other hand, the higher level of n6 PUFAs in cirrhotic tissues was surprising. The levels of two examples of this FA group, n6 docosapentaenoic acid (DPA n6) and adrenic acid (AdA), were significantly increased in damaged liver samples (Figure 9).

**Figure 10:**
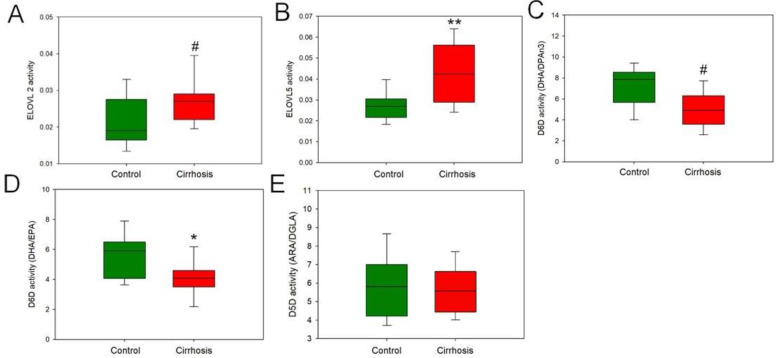
Estimated activities of enzymes ELOVL2 (**A**), ELOVL5 (**B**), D6D (DHA/DPAn3) (**C**), D6D (DHA/EPA) **(D**) and D5D (**E**) in the cirrhosis livers and hepatic control. For parametric values: Student’s *t*-test * *p* < 0.05; ** *p* < 0.001; for non-parametric: Mann–Whitney U test # *p* < 0.05.

The estimated enzyme activities (EEAs) of FAs in the livers were calculated as the ratio of product to substrate for ELOVL 5/AdA/ARA; for ELOVL 2/DPAn6/ARA; for desaturase 5 (D5D)/ARA/dihomo-γ-linolenic acid (DGLA); for D6D–DHA/EPA; and for D6D–DHA/DPAn3. The estimated EEAs confirmed the results of the GC-MS analysis. Higher ELOVL5 and ELOVL2 activity levels, responsible for AdA and DPAn6 formation, respectively, were observed in cirrhotic tissues compared to the liver control (Figure 10A,B). In turn, the activity of D6D, a lipogenic enzyme responsible for DHA synthesis, decreased in cirrhotic samples (Figure 10C,D). The next lipogenic enzyme D5D, which is responsible for ARA synthesis, did not change in the liver samples examined (Figure 10E). The ARA level also did not differ between the two liver groups examined (Supplementary Table S1).

### 2.2. Effect of Cirrhosis on Hepatic FA Metabolism

**Figure 11:**
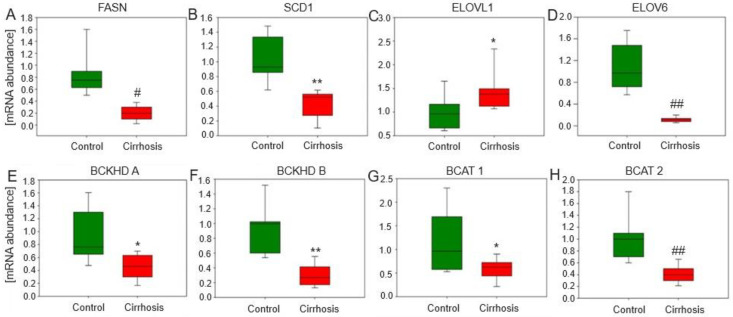
Relative expression level of genes involved in lipid synthesis in liver tissue. FASN: fatty acid synthetase; SCD1: stearoyl-CoA desaturase-1; ELOVL1: fatty acid elongase 1; ELOVL6: fatty acid elongase 6; BCKHD A: branched-chain alpha-keto acid dehydrogenase A, BCKHD B: branched-chain alpha-keto acid dehydrogenase B, BCAT1: branched chain amino acid transaminase 1, BCAT2: branched chain amino acid transaminase 2. For parametric values: Student’s *t*-test * *p* < 0.05; ** *p* < 0.001; for non-parametric: Mann–Whitney U test # *p* < 0.05; ## *p* < 0.001.

Gene expression analysis performed in the cirrhotic tissue samples showed lower expression of almost all genes tested. The only exception to this rule was ELOVL1-fatty acid elongase 1, whose expression was higher in cirrhotic livers (Figure 11). Furthermore, all the differences in mRNA abundance reached statistical significance, which is a good indication of the group trend, despite the rather large statistical variation in the results of individual patients.

**Figure 12:**
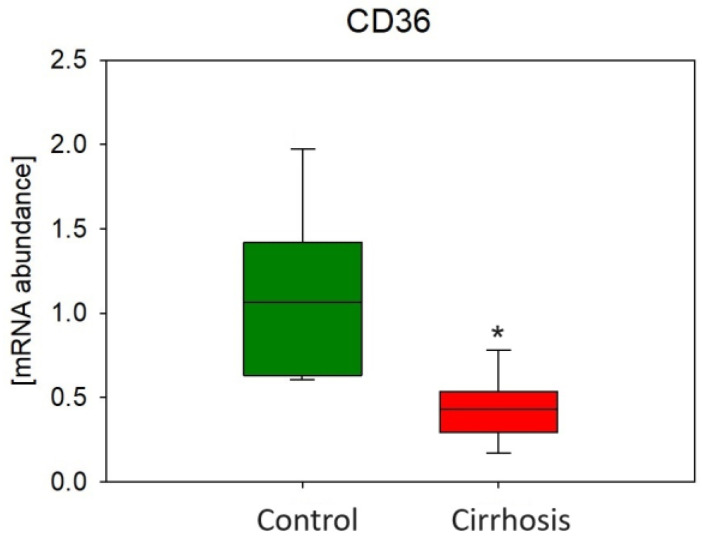
Relative expression level of fatty acid translocase (CD36). * *p* < 0.05.

To check if FA transport may be disturbed in cirrhotic livers, we analyzed the mRNA level of FA transporter CD36. In the tissues of patients with cirrhosis, we found a statistically significant decrease in the expression of the CD36 gene, the encoding FA transporter, compared to the group without cirrhosis (Figure 12).

**Figure 13:**
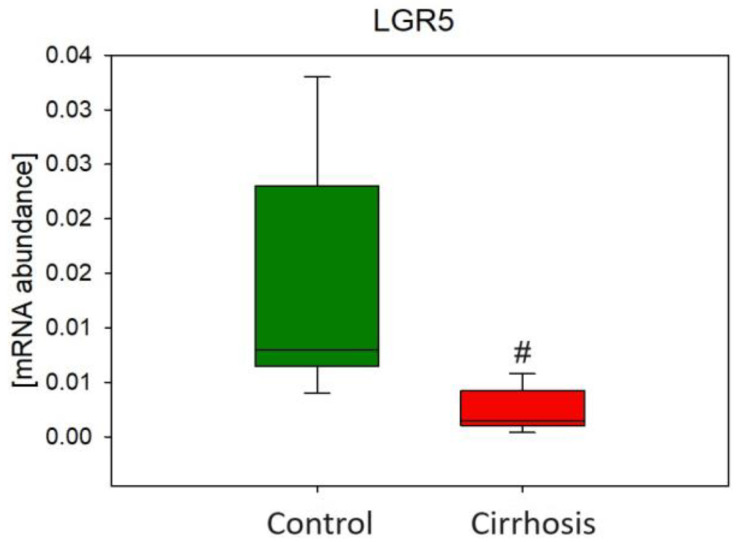
Relative expression level of leucine-rich repeat-containing G-protein coupled receptor 5 (LGR5). Mann–Whitney U test # *p* < 0.05.

Additionally, to verify the possible relationship between liver cells expressing leucine-rich repeat-containing G-protein coupled receptor 5 (LGR5) and the process of liver cirrhosis, we measured the mRNA level of gene encoding this protein. We found a significantly lower mRNA level of LGR5 in cirrhotic liver tissue (Figure 13).

## 3. Discussion

Exploring the changes in the FA profile and expression of genes related to FA metabolism in cirrhotic liver tissue reveals a molecular landscape that goes beyond the surface of traditional liver function assessments. Understanding the shifts in gene expression and FA composition in liver tissue opens avenues for interventions that may aid in the treatment of cirrhosis in the future.

The results on the changes in the FA profile and the expression of genes related to FA metabolism in cirrhotic liver tissue offer some insights into the molecular landscape of this severe liver disease. In interpreting the results, several key themes emerged that shed light on the potential impact of the pathogenesis and progression of cirrhosis.

Our observation of increased SFAs and reduced n3 PUFAs in cirrhotic liver tissue is consistent with most studies of damaged liver tissue, indicating a distinct molecular signature associated with this pathologic condition [13,14]. The shift towards SFAs could contribute to a pro-inflammatory environment in the liver and possibly even exacerbate tissue damage [14,15,16]. At the same time, the lower levels of n3 PUFAs, which are crucial for the anti-inflammatory response and homeostasis in cells, suggest that the liver is unable to cope with ongoing oxidative stress [17,18].

The identification of elevated levels of VLC-MUFAs, VLC-SFAs, and C16:1 in patients with cirrhosis represents a significant and potentially worrying aspect of our findings. These FAs have been associated with a high cardio-metabolic risk in several studies published prior to our study [19,20,21]. MUFAs are also thought to be associated with conditions such as T2D and IR [22,23], which are becoming an epidemic problem worldwide. However, there are a lot of mixed data on the beneficial and destructive role of MUFA-enriched food [21,24,25,26], so we cannot determine their influence on the structure of damaged liver tissue. In addition, understanding the specific role of VLCFAs in the context of cirrhosis may reveal new insights into the pathophysiology of the disease and the altered metabolism of liver cells [18].

The decreased BCFA content in cirrhotic patients raises concerns about possible unfavorable consequences. BCFAs, known for their anti-inflammatory properties, have been associated with some health benefits [27,28]. The reduction in these beneficial FAs in cirrhosis could also contribute to liver inflammation.

In conclusion, our observation of increased VLC-MUFAs, VLC-SFAs, and C16:1 alongside decreased BCFAs in cirrhotic patients underscores the complexity of the metabolic changes in this disease. Future studies investigating the relationships between different FAs and clinical markers in cirrhotic patients should definitely be considered.

The question arises whether the altered FA profiles in cirrhotic liver tissue are in any way correlated with dysregulated gene expression. In different tissues and under different conditions, genes involved in the oxidation, synthesis, and transport of FAs show significant changes, suggesting a link between genes and metabolic shifts in lipid metabolism [29,30,31]. Understanding these genetic patterns may contribute to the understanding of the molecular mechanisms that lead to the changes in the FA profile.

The downregulation of almost all analyzed genes (FASN, SCD1, ELOVL6, BCKDHA, BCKDHB, BCAT1, BCAT2, and CD36) in the cirrhotic liver tissue samples in this study was a surprising observation. A study previously performed in a rodent model [32] showed that enzymes responsible for the desaturation and synthesis of FAs have even higher levels of RNA in the tissues of animals that consumed alcohol. Moreover, FASN expression was elevated in guinea pigs with liver fibrosis [33]. Considering this fact, we expected that the mRNA levels of the analyzed genes were increased compared to the hepatic control samples. This significant reduction in the expression of various genes may be the result of a regulatory failure in the damaged liver or simply fibrosis of the liver tissue [34]. In contrast, patients with NAFLD have an increased expression of genes, such as acetyl-CoA carboxylase (ACC1), FASN, and SCD1 [5,32]. Therefore, we can hypothesize that the observed FA changes may be the result of processes that were active at earlier stages of the disease. Some data can support our results. For example, mice with the SCD1 knockout have increased liver fibrosis and increased markers for liver cirrhosis (eg. TGFb, and collagen) [35]. This suggests that decreased SCD1 expression may not only be a result of liver tissue damage but also may contribute to the progression of fibrosis. Another study’s results also match with ours, showing a lower activity of BCKHD in cirrhotic human livers compared to control liver tissues [36]. In contrast to other studied genes, we found that ELOVL1 gene expression was upregulated in cirrhotic liver tissue. Our results are consistent with those of Kyrytsi et al. [37], who also found increased ELOVL1 protein levels in livers of patients with cirrhosis suffering from alcoholism compared to control subjects. Considering the elevated levels of VLCFA and VLC-MUFA, we can speculate that the expression of ELOVL1 remained at an elevated level despite liver tissue damage and was probably even higher in earlier stages of the disease. Investigating the metabolic consequences of ELOVL1 overexpression may reveal a new therapeutic target. We strongly believe that understanding the reasons behind the upregulation of ELOVL1 and VLCFA could give us some insights into the molecular pathways of liver cirrhosis.

Additionally, we measured LGR5 gene expression to see if this protein—which is a marker of adult stem cells in various tissues, including the liver—may also influence the process of liver cirrhosis. The hepatocytes that express the LGR5 gene can repair liver tissues that have been exposed to damage because they promote the regeneration of hepatocytes and ductal cells [38]. Thus, the decreased expression of LGR5 in cirrhotic liver may contribute to the development of liver damage in our patients.

Our study has several advantages. Due to our collaboration with a surgical department team, we were able to obtain tissue samples from cirrhotic livers and control tissues in the middle of a surgery. In this respect, our experimental design appears to be completely unique and unfathomable. Our study has also certain limitations, including a relatively small sample size. Moreover, the study group consisted mainly of patients with ALD, which is not a major focus point worldwide. In addition, it is possible that the lipid composition of the liver in the control group who underwent liver tumor resection was different from that of healthy individuals without liver impairment. Nevertheless, our different results reached statistical significance.

## 4. Materials and Methods

### 4.1. Subjects

The study group included 21 patients (6 women and 15 men) diagnosed with cirrhosis, who underwent orthotopic liver transplantation in the Department of General, Endocrine, and Transplant Surgery at the Medical University of Gdansk (Poland). Our patients suffered from cirrhosis with different etiological backgrounds, such as ALD, HBV, or HCV infection; NASH; autoimmune hepatitis; primary sclerosing cholangitis; and MAFLD. The control group consisted of 16 patients (8 women and 8 men), who had undergone liver tumor resection. Blood was collected from the patients before surgery. The laboratory parameters were determined in the patients’ blood at the Central Clinical Laboratory, Medical University of Gdansk. Selected biochemical and anthropometric parameters of the two groups are presented in Table 1.

### 4.2. Sample Collection

Liver biopsies were taken from both the livers with cirrhosis and the hepatic control group. Written informed consent was obtained before the procedure in each case. In patients from the cirrhotic liver group, the cirrhotic liver was removed during orthotropic liver transplantation–hepatectomy. At an early stage of the procedure, when the blood flowing through the liver tissue was still present, a representative biopsy was taken. The liver parenchyma was transfected, and a sample was taken from the sectional plane of the left lobe of the liver. The sample was taken at least 1 cm deep from the surface to avoid distortions related to the fibrosis process of the liver capsule. Patients from the control group: in the anatomical resection of a liver tumor from a non-cirrhotic liver, an entire segment or segments of the liver was removed. In the early phase of resection, after the initial parenchymal transection, a sample was taken (always from the area similar to the cirrhotic group): 1 cm deep from the surface, from the incision plane of the removed liver segment. A patient was excluded from this study if taking a sample affected the radicality of the resection or influenced the final histopathologic examination. More than 500 mg of each liver biopsy sample was collected. Samples were then immediately frozen in liquid nitrogen and stored at −80 °C until analysis.

### 4.3. Sample Preparation

Total lipids were extracted from the liver tissue samples using a mixture of chloroform/methanol 2:1 (*v*/*v*), as described in Folch et al. [39]. The lipid extracts were evaporated to dryness under nitrogen stream and then weighed on an analytical scale. The samples were hydrolyzed with 1 mL of 0.5 KOH in methanol for 3 h at 90 °C. Then, 0.2 mL of 6 M HCl was added, and the non-esterified FAs were extracted three times with H_2_O and n-hexane and dried by evaporation under nitrogen steam. The FA methyl esters (FAMEs) were obtained by incubation with 1 mL of 10% BF3 in methanol for 1.5 h at 55 °C. Subsequently, 1 mL of H_2_O was added to mixture, and the FAMEs were re-extracted three times with n-hexane. The dried samples were stored at −20 °C until analysis.

### 4.4. Gas Chromatography–Mass Spectrometry Analysis

The analysis of FAMEs was performed using a GC-EI-MS QP-2010SE (Shimadzu, Kyoto, Japan) with Zebron ZB-5MSi column of 30 m length × 0.25 µm i.d. × 0.25 µm film thickness (Phenomenex, Torrance, CA, USA). The GC oven was set to 60–300 °C (increase of 4 °C per minute), with a total analysis time of 60 min. Helium was used as the carrier gas, and the column head pressure was set at 100 kPa. The electron energy of 70 eV was used to FAME ionization. 19-methylarachidic acid was used as an internal standard. Full scan mode analysis with mass scan range *m*/*z* 45–700 was applied. Accurate identification of the FA profile was possible based on FAME mixture standards (Larodan, Monroe, MI, USA, and Merck, Darmstadt, Germany).

### 4.5. Analysis of mRNA Level

Total RNA was extracted from deep frozen tissue samples using RNeasy Plus Universal Mini Kit (Qiagen, Venlo, The Netherlands) according to the attached protocol. The RNA was eluted through the elution column with 50 µL of RNAse and DNAse-free water. First, the RNA concentration and two purity ratios—260/230 and 260/280—were determined using NanoDrop™ One/OneC (Thermo Fisher Scientific, Waltham, MA, USA). Samples that achieved an absorbance between 1.6 and 2.2 were used for gene expression analysis. Furthermore, the samples were analyzed again for quality and RNA integrity using automated gel electrophoresis (Experion, Bio-Rad, Hercules, CA, USA). Only the samples with an RQI above 6.0, reflecting the representative RNA, were considered. We then synthesized cDNA from total RNA after enzymatic digestion of DNA using the RevertAid First Strand cDNA Synthesis Kit (Thermo Fisher Scientific, Waltham, MA, USA) and diluted it at a ratio of 1/25 with RNase-free water. The mRNA concentrations for gene expression analysis were determined by real-time PCR, using the CFX Connect Real-Time System (Bio-Rad) with SensiFAST SYBR NO-ROX Kit (Thermo Fisher Scientific, USA) detection. Human cyclophilin was used as a housekeeping gene in all of the analyzed samples (sequence forward—CGTCTCCTTTGAGCTGT, reverse—TCGAGTTGTCCACAGTCA). The primer sequences used for all genes selected for our analysis were as follows: FASN–forward CTCGTTGAAGAACGCATCCA, reverse CGCTCGGCATGGCTATCT; SCD1–forward AACAGTGTGTTCGTTGCCACTT, reverse GGTAGTTGTGGAAGCCCTC; ELOVL1–forward CTGTGGCACAACCCTACCTT, reverse CTGGGAGATGTGCAGTGAGA; ELOVL6–forward CAAAGCACCCGAACTAGGAG, reverse TGGTGATACCAGTGCAGGAA; FADS1–forward CCAACTGCTTCCGCAAAGAC, reverse GCTGGTGGTTGTACGGCATA; FADS2–forward AAGGGTGCCTCTGCCAACT, reverse GATTGTAGGGCAGGTATTTCAGC; BCKDHA–forward GATGACAAGCCCCAGTTCCCA, reverse TGGGGTTGATGATCTGGCCTT; BCKDHB–forward GCGGCAGGTGGCTCATTTTACT, reverse CAGTAGGATCTTTGGCCAATGAGTTAT; BCAT1–forward GGTCCCATATTCAACATCTGCTAGTCT, reverse TCCCATCTTGCAGTCCCCAGT; BCAT2–forward TTACGCGCCGCACGGATCAT, reverse GGTCGGTAAATGTCTTCCCAAAC; CD36–forward AAGTCACTGCGACATGATTAATGG, reverse GAACTGCAATACCTGGCTTTTCTC; LGR 5–forward CCTGCTTGACTTTGAGGAAGACC, reverse CCAGCCATCAAGCAGGTGTTCA. The amplification of the specific transcripts was confirmed using melting curve profiles in comparison to the profiles of the housekeeping gene.

### 4.6. Analysis of Hydroxyproline Content

Hydroxyproline analysis of the collected liver tissue samples was performed using a specialized kit (Merck, Darmstadt, Germany). In brief, tissue sections of the appropriate weight were homogenized and then hydrolyzed in 10 M NaOH. Samples were then heated at 120 °C for one hour, neutralized with 10 M HCl, and centrifuged at 10,000× g for 5 min to remove all impurities. The remaining supernatant was used to determine the hydroxyproline concentration in the sample using a colorimetric assay according to the manufacturer’s instructions. The analyzed platelets were read with the Synergy HT Microplate Reader (BioTek, Winooski, VT, USA) at a wavelength of 560 nm.

### 4.7. Statistical Analysis

Data analysis was performed in SigmaPlot 14.5 (Systat Software Inc., San Jose, CA, USA). All values are given as mean ± SD. A value of *p* < 0.05 was considered statistically significant. The significance of differences between FA profiles and gene expression levels in cirrhotic and hepatic control tissues was tested using Student’s *t*-test for the parametric distribution (* *p* < 0.05, ** *p* < 0.001) and the Mann–Whitney test for the non-parametric distribution (# *p* < 0.05, ## *p* < 0.001). The correlation was calculated using the Pearson correlation coefficient. The relevant expression levels were determined by the 2^−ΔΔ^ formula. 

## Figures and Tables

**Table 1:** Clinical and anthropomorphic characteristics of groups.

Parameter	Hepatic Control	Patients with Cirrhosis	*p* Value
Age (years)	57.9 ± 17.6	50.6 ± 11.3	NS
BMI (kg/m^3^)	27.5 (23.5; 32.0)	26.0 (23.5; 32.5)	NS
MELD-Na	7.00 ± 1.63	17.0 ± 7.27	0.012 *
Total cholesterol (mg/dL)	151 ± 38.1	138 ± 50.9	NS
LDL-C (mg/dL)	80.9 ± 27.9	90.0 ± 40.3	NS
HDL-C (mg/dL)	51.2 ± 15.2	35.0 ± 13.3	0.017 *
TAG (mg/dL)	98.0 ± 43.9	91.7 ± 24.7	NS
Glucose (mg/dL)	96.0 (89.5; 110)	101 (93.0; 116)	NS
Bilirubin (mg/dL)	0.58 (0.40; 0.75)	2.0 (1.16; 4.05)	<0.001 ##
GGTP (IU/L)	43.0 (24.0; 96.0)	79.0 (47.0; 238)	NS
ALT (U/L)	20.0 (17.0; 41.0)	36.0 (13.0; 72.0)	NS
AST (U/L)	25.5 (19.0; 37.8)	51.0 (28.5; 90.5)	0.033 #
WBC (109/l)	7.37 (5.66; 8.87)	5.46 (3.56; 7.54)	0.022 #
ALP (U/L)	76.0 (66.5; 113)	116 (91.3; 218)	0.034 #
Creatinine (mg/dL)	0.81 (0.69; 0.94)	0.82 (0.67; 1.25)	NS
Albumin (g/L)	38.6 ± 3.75	33.2 ± 6.81	0.028 *

For parametric: values presented as mean ± SD; Student’s *t*-test * *p* < 0.05;NS—not significant. For non-parametric: values presented as median and 25% and 75% quartiles; Mann–Whitney U test # *p* < 0.05; ## *p* < 0.001. ALT—alanine aminotransferase, ALP—alkaline phosphatase, AST—asparagine aminotransferase, BMI—body mass index, GGTP—gamma-glutamyl transpeptidase, HDL-C—high-density lipoprotein cholesterol, LDL-C—low-density lipoprotein cholesterol, MELD-Na—Model of End-Stage Liver Disease for liver cirrhosis, TAG—triacylglycerols, WBC—white blood cells.

## Data Availability

Data is contained within the article and supplementary material.

## Supplementary Materials

The following supporting information can be downloaded at: https://www.mdpi.com/article/10.3390/ijms25158115/s1.

## References

[1] Ginès P., Krag A., Abraldes J.G., Solà E., Fabrellas N., Kamath P.S. (2021). Liver cirrhosis. Lancet.

[2] Singal A.K., Bataller R., Ahn J., Kamath P.S., Shah V.H. (2018). ACG Clinical Guideline: Alcoholic Liver Disease. Am. J. Gastroenterol..

[3] Liu S.Y., Tsai I.T., Hsu Y.C. (2021). Alcohol-related liver disease: Basic mechanisms and clinical perspectives. Int. J. Mol. Sci..

[4] Yan C., Hu W., Tu J., Li J., Liang Q., Han S. (2023). Pathogenic mechanisms and regulatory factors involved in alcoholic liver disease. J. Transl. Med..

[5] Hliwa A., Ramos-Molina B., Laski D., Mika A., Sledzinski T. (2021). The role of fatty acids in non-alcoholic fatty liver disease progression: An update. Int. J. Mol. Sci..

[6] Younossi Z.M., Golabi P., Paik J.M., Henry A., Van Dongen C., Henry L. (2023). The global epidemiology of nonalcoholic fatty liver disease (NAFLD) and nonalcoholic steatohepatitis (NASH): A systematic review. Hepatology.

[7] Eslam M., Newsome P.N., Sarin S.K., Anstee Q.M., Targher G., Romero-Gomez M., Zelber-Sagi S., Wai-Sun Wong V., Dufour J.F., Schattenberg J.M. (2020). A new definition for metabolic dysfunction-associated fatty liver disease: An international expert consensus statement. J. Hepatol..

[8] Nguyen P., Leray V., Diez M., Serisier S., Le Bloc’H J., Siliart B., Dumon H. (2008). Liver lipid metabolism. J. Anim. Physiol. Anim. Nutr..

[9] Puri P., Baillie R.A., Wiest M.M., Mirshahi F., Choudhury J., Cheung O., Sargeant C., Contos M.J., Sanyal A.J. (2007). A lipidomic analysis of nonalcoholic fatty liver disease. Hepatology.

[10] Yamada K., Mizukoshi E., Sunagozaka H., Arai K., Yamashita T., Takeshita Y., Misu H., Takamura T., Kitamura S., Zen Y. (2015). Characteristics of hepatic fatty acid compositions in patients with nonalcoholic steatohepatitis. Liver Int..

[11] Pawlosky R.J., Salem N. (2004). Perspectives on alcohol consumption: Liver polyunsaturated fatty acids and essential fatty acid metabolism. Alcohol.

[12] Gabr S.A., Alghadir A.H., Sherif Y.E., Ghfar A.A. (2016). Hydroxyproline as a Biomarker in Liver Disease. Expo. Health.

[13] Mozaffarian D., Wu J.H.Y. (2011). Omega-3 fatty acids and cardiovascular disease: Effects on risk factors, molecular pathways, and clinical events. J. Am. Coll. Cardiol..

[14] Sehgal R., Perfilyev A., Männistö V., Ågren J., Nilsson E., Käkelä P., Ling C., de Mello V.D., Pihlajamäki J. (2023). Liver saturated fat content associates with hepatic DNA methylation in obese individuals. Clin. Epigenet..

[15] Rada P., González-Rodríguez Á., García-Monzón C., Valverde Á.M. (2020). Understanding lipotoxicity in NAFLD pathogenesis: Is CD36 a key driver?. Cell Death Dis..

[16] Lundbom J., Hakkarainen A., Söderlund S., Westerbacka J., Lundbom N., Taskinen M.R. (2011). Long-TE 1H MRS suggests that liver fat is more saturated than subcutaneous and visceral fat. NMR Biomed..

[17] Yan P., Luo Y., Huang Z., Mou T., Yang H., Peng D., Wu Z. (2023). Establishment of a prognostic signature based on fatty acid metabolism genes in HCC associated with hepatitis B. BMC Gastroenterol..

[18] Zhang H., Axinbai M., Zhao Y., Wei J., Qu T., Kong J., He Y., Zhang L. (2023). Bioinformatics analysis of ferroptosis-related genes and immune cell infiltration in non-alcoholic fatty liver disease. Eur. J. Med. Res..

[19] Kris-Etherton P.M. (1999). Monounsaturated Fatty Acids and Risk of Cardiovascular Disease AHA Science Advisory. Circulation.

[20] Sarabhai T., Kahl S., Szendroedi J., Markgraf D.F., Zaharia O.P., Barosa C., Herder C., Wickrath F., Bobrov P., Hwang J.H. (2020). Monounsaturated fat rapidly induces hepatic gluconeogenesis and whole-body insulin resistance. JCI Insight.

[21] Imamura F., Lemaitre R.N., King I.B., Song X., Steffen L.M., Folsom A.R., Siscovick D.S., Mozaffarian D. (2013). Long-chain monounsaturated fatty acids and incidence of congestive heart failure in 2 prospective cohorts. Circulation.

[22] Gillingham L.G., Harris-Janz S., Jones P.J.H. (2011). Dietary monounsaturated fatty acids are protective against metabolic syndrome and cardiovascular disease risk factors. Lipids.

[23] Jiang S., Yang W., Li Y., Feng J., Miao J., Shi H., Xue H. (2023). Monounsaturated and polyunsaturated fatty acids concerning prediabetes and type 2 diabetes mellitus risk among participants in the National Health and Nutrition Examination Surveys (NHANES) from 2005 to March 2020. Front. Nutr..

[24] Schwingshackl L., Hoffmann G. (2014). Monounsaturated fatty acids, olive oil and health status: A systematic review and meta-analysis of cohort studies. Lipids Health Dis..

[25] Yang Z.H., Miyahara H., Iwasaki Y., Takeo J., Katayama M. (2013). Dietary supplementation with long-chain monounsaturated fatty acids attenuates obesity-related metabolic dysfunction and increases expression of PPAR gamma in adipose tissue in type 2 diabetic KK-Ay mice. Nutr. Metab..

[26] Bozzetto L., Prinster A., Annuzzi G., Costagliola L., Mangione A., Vitelli A., Mazzarella R., Longobardo M., Mancini M., Vigorito C. (2012). Liver Fat Is Reduced by an Isoenergetic MUFA Diet in a Controlled Randomized Study in Type 2 Diabetic Patients. Diabetes Care.

[27] Gozdzik P., Magkos F., Sledzinski T., Mika A. (2023). Monomethyl branched-chain fatty acids: Health effects and biological mechanisms. Prog. Lipid Res..

[28] Yan Y., Wang Z., Greenwald J., Kothapalli K.S.D., Park H.G., Liu R., Mendralla E., Lawrence P., Wang X., Brenna J.T. (2017). BCFA suppresses LPS induced IL-8 mRNA expression in human intestinal epithelial cells. Prostaglandins. Leukot. Essent. Fatty Acids.

[29] Jeyakumar S.M., Vajreswari A. (2022). Stearoyl-CoA desaturase 1: A potential target for non-alcoholic fatty liver disease?—Perspective on emerging experimental evidence. World J. Hepatol..

[30] Du T.Y., Gao Y.X., Zheng Y.S. (2023). Identification of key genes related to immune infiltration in cirrhosis via bioinformatics analysis. Sci. Rep..

[31] Paul B., Lewinska M., Andersen J.B. (2022). Lipid alterations in chronic liver disease and liver cancer. JHEP Rep..

[32] You M., Fischer M., Deeg M.A., Crabb D.W. (2002). Ethanol Induces Fatty Acid Synthesis Pathways by Activation of Sterol Regulatory Element-binding Protein (SREBP). J. Biol. Chem..

[33] Ipsen D.H., Skat-Rørdam J., Tsamouri M.M., Latta M., Lykkesfeldt J., Tveden-Nyborg P. (2019). Molecular drivers of non-alcoholic steatohepatitis are sustained in mild-to-late fibrosis progression in a guinea pig model. Mol. Genet. Genom..

[34] Samarasinghe S.M., Hewage A.S., Siriwardana R.C., Tennekoon K.H., Niriella M.A., De Silva S. (2023). Genetic and metabolic aspects of non-alcoholic fatty liver disease (NAFLD) pathogenicity. Egypt. J. Med. Hum. Genet..

[35] Cootway D., Kalyesubula M., Miller J., Huff H., Lefers L., Christofi V.P., Anderson E., Ntambi J. (2024). SCD1 deficiency promotes hepatic fibrosis and cirrhosis under a high carbohydrate low-fat diet. J. Biol. Chem..

[36] Shimomura Y., Honda T., Goto H., Nonami T., Kurokawa T., Nagasaki M., Murakami T. (2004). Effects of liver failure on the enzymes in the branched-chain amino acid catabolic pathway. Biochem. Biophys. Res. Commun..

[37] Kyritsi K., Wu N., Zhou T., Carpino G., Baiocchi L., Kennedy L., Chen L., Ceci L., Meyer A.A., Barupala N. (2023). Knockout of secretin ameliorates biliary and liver phenotypes during alcohol-induced hepatotoxicity. Cell Biosci..

[38] Han J., Lin K., Zhang X., Yan L., Chen Y., Chen H., Liu J., Liu J., Wu Y. (2021). PTEN-mediated AKT/β-catenin signaling enhances the proliferation and expansion of Lgr5+ hepatocytes. Int. J. Biol. Sci..

[39] Folch J., Lees M., Sloane Stanley G.H. (1957). A simple method for the isolation and purification of total lipides from animal tissues. J. Biol. Chem..

