# Supplementary material for: Alterations of the Fatty Acid Profile and the Expression of Genes Related to FA Metabolism in Cirrhotic Liver Tissue
Source: Int J Mol Sci. 2024 Jul 25;25(15):8115. doi: 10.3390/ijms25158115 (PMC11311876; doi:10.3390/ijms25158115)
Supplement: Supplementary file 1 [file ijms-25-08115-s001.zip › ijms-3079323-supplementary.pdf]

Table S1. Comparison of other detected fatty acids between patients with cirrhosis and hepatic control group.

FA	Hepatic group	Cirrhosis group	p value
**C12**	0.20 ± 0.056	0.23 ± 0.032	NS
**C14**	1.39 ± 0.158	1.63 ± 0.143	NS
**C16**	24.3 ± 0.54	24.5 ± 0.66	NS
**C18**	12.3 ± 0.57	11.9 ± 0.44	NS
**C26**	0.018 ± 0.002	0.020 ± 0.002	NS
**C28**	0.010 ± 0.000	0.010 ± 0.000	NS
**C13**	0.014 ± 0.002	0.016 ± 0.002	NS
**C15**	0.34 ± 0.021	0.33 ± 0.024	NS
**C19**	0.029 ± 0.001	0.030 ± 0.002	NS
**C21**	0.016 ± 0.002	0.022 ± 0.003	NS
**C23**	0.072 ± 0.007	0.068 ± 0.006	NS
**C25**	0.017 ± 0.002	0.022 ± 0.003	NS
**2,6,10-M-12:0**	0.017 ± 0.002	0.020 ± 0.002	NS
**4,8,12-M-14:0**	0.014 ± 0.002	0.016 ± 0.002	NS
**iso 12-M-13:0**	0.013 ± 0.001	0.012 ± 0.001	NS
**iso 13-M-14:0**	0.027 ± 0.003	0.022 ± 0.003	NS
**iso 20-M-21:0**	0.010 ± 0.000	0.016 ± 0.003	NS
**anteiso 12-M-14:0**	0.051 ± 0.006	0.052 ± 0.005	NS
**anteiso 16-M-18:0**	0.033 ± 0.002	0.034 ± 0.003	NS
**anteiso20-M-22:0**	0.011 ± 0.001	0.014 ± 0.001	NS
**C14:1**	0.068 ± 0.013	0.089 ± 0.012	NS
**C18:1**	26.5 ± 1.36	26.5 ± 0.84	NS
**C19:1**	0.022 ± 0.002	0.020 ± 0.002	NS
**CPOA2H**	0.10 ± 0.014	0.10 ± 0.006	NS

**HDA**	0.019 ± 0.002	0.017 ± 0.002	NS
**LA**	14.9 ± 0.55	13.9 ± 0.56	NS
**EDA**	0.17 ± 0.017	0.17 ± 0.015	NS
**DGLA**	1.39 ± 0.141	1.39 ± 0.101	NS
**ARA**	7.65 ± 0.65	7.73 ± 0.55	NS
**PUFA n6**	24.5 ± 1.31	23.7 ± 0.93	NS
**ALA**	0.13 ± 0.010	0.11 ± 0.011	NS
**ETA**	0.041 ± 0.005	0.044 ± 0.004	NS
**EPA**	0.66 ± 0.089	0.62 ± 0.066	NS
**DPAn3**	0.51 ± 0.048	0.52 ± 0.038	NS
**ECFA**	38.7 ± 0.53	38.9 ± 0.67	NS
**ANSTEISO BCFA**	0.21 ± 0.02	0.18 ± 0.01	NS
**TOTAL SFA**	40.0 ± 0.54	40.1 ± 0.69	NS
**MUFA**	30.7 ± 1.60	32.2 ± 0.94	NS

Mean ± SD, content [%], ALA: α-linolenic acid; ARA: arachidonic acid; BCFA: branch chained fatty acid; DGLA: dihomo-γ-linolenic acid; DHA: docosahexaenoic acid; DPAn3: docosapentaenoic acid n3; ECFA: even chained fatty acid; EDA: eicosadienoic acid; EPA: eicosapentaenoic acid; ETA: eicosatetraenoic acid; HDA: hexadecadienoic acid; LA: linoleic acid; OCFA: odd chained fatty acid; n6 PUFA: n6 polyunsaturated fatty acids; SFA: saturated fatty acids.
